# Commercial Screen-Printed Electrodes Based on Carbon Nanomaterials for a Fast and Cost-Effective Voltammetric Determination of Paracetamol, Ibuprofen and Caffeine in Water Samples

**DOI:** 10.3390/s19184039

**Published:** 2019-09-19

**Authors:** Núria Serrano, Òscar Castilla, Cristina Ariño, M. Silvia Diaz-Cruz, José Manuel Díaz-Cruz

**Affiliations:** 1 Department of Chemical Engineering and Analytical Chemistry, University of Barcelona. Martí i Franquès 1-11, E08028 Barcelona, Spain; ocastilla93@gmail.com (Ò.C.); cristina.arino@ub.edu (C.A.); josemanuel.diaz@ub.edu (J.M.D.-C.); 2 Institut de Recerca de l’Aigua (IdRA), University of Barcelona, E08028 Barcelona, Spain; 3 Department of Environmental Chemistry, Institute of Environmental Assessment and Water Research (IDAEA), Spanish Council of Scientific Research (CSIC), Jordi Girona 18-26, 08034-Barcelona, Spain; sdcqam@cid.csic.es

**Keywords:** screen-printed electrodes, carbon nanomaterials, voltammetry, ibuprofen, paracetamol, caffeine

## Abstract

Carbon screen-printed electrode (SPCE), multi-walled carbon nanotubes modified screen-printed electrode (SPCNTE), carbon nanofibers modified screen-printed electrode (SPCNFE), and graphene modified screen-printed electrode (SPGPHE) were in a pioneer way tested as sensors for the simultaneous determination of the two most consumed pain-killers, paracetamol (PA) and ibuprofen (IB), and the stimulant caffeine (CF) in water by differential pulse voltammetry (DPV). Their analytical performances were compared, and the resulting sensitivities (2.50, 0.074, and 0.24 μA V mg^−1^ L for PA, IB, and CF, respectively), detection limits (0.03, 0.6, and 0.05 mg L^−1^ for PA, IB, and CF, respectively) and quantification limits (0.09, 2.2, and 0.2 mg L^−1^ for PA, IB, and CF, respectively) suggested that the SPCNFE was the most suitable carbon-based electrode for the voltammetric determination of the selected analytes in water at trace levels. The methodology was validated using both spiked tap water and hospital wastewater samples. The results were compared to those achieved by liquid chromatography–tandem mass spectrometry (LC-MS/MS), the technique of choice for the determination of the target analytes.

## 1. Introduction

In recent years, the presence in the environment of trace amounts of emerging contaminants, especially drug residues, in water has been an area of major concern for the general public and the health authorities [1]. The excretion of drugs and their metabolites in combination with inadequate waste disposal and incomplete removal by current wastewater treatments have led to the presence of drug residues in surface water, groundwater, or sludges at concentrations ranging from a few ng L^−1^ to a low mg L^−1^ [2]. As a consequence, these compounds may even enter drinking water produced from groundwater, as has been demonstrated in numerous studies [3]. Thousands of tons of pharmaceuticals are consumed every year to address different diseases. In particular, paracetamol (PA), also known as acetaminophen, 4-acetamidophenol, N-acetyl-p-aminophenol, or tylenol, is an analgesic and antipyretic drug typically used for fever reduction and pain relief associated with headache, backache, postoperative pain, and arthritis [4]. Ibuprofen (IB), denoted chemically as (RS)-2-(4-(2-methylpropyl)phenyl)propanoic acid, is a nonsteroidal anti-inflammatory drug widely used for the treatment of pain, fever, and inflammation caused by menstrual cramps, migraines, and rheumatoid arthritis [4]. Caffeine (CF) (1,3,7-trimethylxanthine) is a central nervous system stimulant of the methylxanthine class, which is the most widely used psychoactive substance in the world. CF is present in coffee, tea, soft drinks, chocolate, cocoa, and numerous prescription and over-the-counter drugs. CF intake may protect against some diseases such as Parkinson’s disease, although it can also produce a mild form of drug dependence when an individual stops consuming it after repeated daily intake. This stimulant is often used in combination with other analgesics to increase their effects [4].

The analysis of drug residues in environmental samples is mainly carried out by gas chromatography (GC) or high-performance liquid chromatography (HPLC) usually in combination with mass spectrometry (MS) [5,6,7]. However, such methods are expensive due to the instrumentation involved and the high quality of the reagents required and need of specialized personnel due to the complexity of the instrumentation, and compared to other analytical methods, generate a lot of waste. Therefore, electroanalytical methods represent an interesting option for drug residues control due to their high sensitivity and selectivity, simplicity, low cost, and capability for on-site analysis [8].

Several papers devoted to the voltammetric individual determination of PA [9,10,11,12,13,14,15,16], IB [17,18,19,20], or CF [21,22,23], or at most the simultaneous determination of two of them [24,25,26,27] were reported in the literature. From these publications, it is worth noting that the determination is mostly carried out in pharmaceutical products or in the case of CF in beverages [23], whereas the voltammetric determination of such analytes in environmental samples has been barely investigated. Moreover, to the best of our knowledge, no publications regarding the simultaneous determination of PA, IB, and CF neither in pharmaceuticals nor in environmental samples were available in the literature.

A wide range of electrodes for the voltammetric determination of PA, IB, and CF have been reported in the literature [8], i.e., mercury electrode, carbon paste electrode, glassy carbon electrode, graphite pencil electrode, boron-doped diamond electrode, or chemically modified electrodes. However, in recent years, screen-printing microfabrication technology stands out allowing the mass fabrication of numerous highly-reproducible single-use screen-printed electrodes (SPEs), which are distinguished for their low-cost and accessibility. Moreover, the use of SPEs where the working electrode surface was modified with nanomaterials, such as carbon nanofibers (CNFs), carbon nanotubes (CNTs), or graphene (GPH), offers a larger electrode surface and enhanced electron transfer improving the analytical performance [28]. Thus, the coupling of electroanalytical methods with disposable SPEs modified with nanomaterials represents an attractive and innovative option for the determination of drug residues.

In the present work, the use of different carbon-based modified SPEs, such as carbon (SPCE), carbon nanofibers (SPCNFE), multi-walled carbon nanotubes (SPCNTE), and graphene (SPGPHE), was analytically compared for the determination of PA, IB, and CF by differential pulse voltammetry (DPV). On the basis of the best performance, the selected electrode was applied in the voltammetric simultaneous determination of PA, IB, and CF in real water samples. The results were compared with those achieved by liquid chromatography - tandem mass spectrometry (LC-MS/MS), the technique usually applied for the determination of the target analytes.

## 2. Materials and Methods

### 2.1. Chemicals and Standard Solutions

CF, IB, PA, maleic acid, and sodium hydroxide were purchased from Sigma-Aldrich (St. Louis, MO, USA). Ammonia, sodium dihydrogen phosphate, sulphuric acid, acetic acid, and sodium acetate were supplied by Merck (Darmstadt, Germany). Absolute ethanol was provided by Panreac (Barcelona, Spain). All reagents used were of analytical grade.

Stock standard solutions of PA and CF at 10^−2^ mol L^−1^ were weekly prepared in ultrapure water (Milli-Q plus 185 system, Millipore), whereas a 10^−2^ mol L^−1^ stock standard solution of IB was prepared in absolute ethanol. All stock solutions were stored in the refrigerator at 4 °C protected from light. Daily standard solutions of PA, IB, and CF were prepared by appropriate dilution of the stock standard solutions in ultrapure water.

A tap water sample was collected in the laboratory from the local water distribution network, managed by Agbar Company (Barcelona, Spain; http://www.agbar.es/eng/home.asp) mostly made up from Llobregat River water. In order to test a real sample with potentially more pharmaceutical residues, a wastewater sample collected in a hospital from Mahdia (Tunisia) was also analysed.

Ultrapure water was used in all experiments.

### 2.2. Apparatus

Differential pulse voltammetric (DPV) measurements were carried out in a VA Stand 663 (Metrohm, Herisau, Switzerland) connected to a computer-controlled potentiostat – Autolab Type III) with GPES version 4.9 data acquisition software (EcoChemie, Utrecht, The Netherlands).

Ag/AgCl/KCl (3 mol L^−1^) and Pt wire (Metrohm, Herisau, Switzerland) were the reference electrode and the auxiliary electrode, respectively. The working electrodes used were four different carbon- based screen-printed disk electrodes with 4 mm diameter purchased from Dropsens (Oviedo, Spain): carbon screen-printed electrodes (ref. 110, DS SPCE), multi-walled carbon nanotubes modified screen-printed electrodes (ref. 110CNT, DS SPCE), carbon nanofibers modified screen-printed electrodes (ref. 110CNF, DS SPCE), and graphene modified screen-printed electrodes (ref. 110GPH, DS SPCE).

Screen-printed electrodes were coupled to the Autolab System using a flexible cable (ref. CAC, DropSens).

pH measurements were performed using a Crison micro pH 2000 (Hach Lange Spain, L’Hospitalet de Llobregat, Spain).

### 2.3. Differential Pulse Voltammetry (DPV) Measurements

Unless otherwise indicated, DPV measurements using carbon-based SPEs were performed scanning the potential from 0.1 V to 1.75 V using a step potential of 5 mV, pulse amplitudes of 100 mV, pulse times of 50 ms, and a scan rate of 10 mV s^−1^.

Separate and simultaneous calibration plots were carried out increasing PA, IB, and/or CF concentrations in 0.1 mol L^−1^ acetate buffer at pH 5.5. Analytical parameters were calculated from two independent calibration curves.

Validation of the methodology was performed using 5.0 mL of the sample (spiked tap water or hospital wastewater) and 0.1 mol L^−1^ acetate buffer (pH 5.5) up to a final volume of 20.0 mL. The resulting solution was placed in the cell and the scan was recorded. Standard addition was used as the calibration method. Three aliquots of standard solutions of PA, IB, and CF (spiked tap water) or only PA (hospital wastewater) were further added and the corresponding curves were recorded.

### 2.4. Liquid Chromatography–Tandem Mass Spectrometry (LC-MS/MS) Measurements

The LC-MS/MS determination of PA, IB, and CF in the wastewater sample was carried out in a HP 1100 chromatograph (Agilent Technologies, Palo Alto, CA, USA) attached to a 4000 QTRAP mass spectrometer (Applied Biosystems, Foster City, CA, USA) equipped with a turbospray electrospray (ESI) interface and was based on the method described in Garcia–Galan et al. [29]. The chromatographic separation was achieved using an Atlantis C18 (Waters, 150 mm × 2.1 mm, 6 µm) LC-column. The mobile phase consisted of HPLC-grade water and acetonitrile, both 0.1% in formic acid. MS/MS data acquisition was performed in the selected reaction monitoring (SRM) mode. Instrument control and data acquisition were carried out using the Analyst 1.4.2 software package Applied Biosystems.

## 3. Results and Discussion

### 3.1. Optimization of Condition Media and the Potential Range

Both potential range and condition media were optimized in connection with the simultaneous determination of PA, IB, and CF by DPV using the conventional SPCE as a carbon based SPE model.

DPV measurements of 20 mg L^−1^ PA, IB, and CF solutions were performed in the presence of different buffers (Figure S1): 0.05 mol L^−1^ sulphuric acid (pH 1), 0.1 mol L^−1^ acetate buffer (pH 4.5), 0.1 mol L^−1^ maleate buffer (pH 6.8), 0.1 mol L^−1^ phosphate buffer (pH 7.4), and 0.1 mol L^−1^ ammonia buffer (pH 8.6). At highly acidic pH values only one peak close to 0.6 V corresponding to PA was detected whereas at pH 6.8, 7.4 and 8.6 a second peak at ca. 1.3 V appeared that could be attributed to CF. In contrast, at pH 4.5 three peaks could be identified; a well-defined peak corresponding to PA, and two overlapped peaks associated to IB and CF. Considering that the acetate buffer was the media allowing the identification of the three target analytes, a more detailed study in acetate buffer solution at pHs from 4.0 to 6.0 was performed (Figure S2). According to this study and considering that p*K*_a_ of the acetic acid is 4.75, the pH value of 5.5 was selected, since it provides the best resolution and peak shape for the three selected analytes. Once the condition media was set up, different potential ranges from 0 to 2.0 V were studied, from 0.1 to 1.75 V being optimal. Thus, in the following experimental work, a potential range from 0.1 to 1.75 V in 0.1 mol L^−1^ acetate buffer at pH 5.5 was applied.

### 3.2. Repeatability and Reproducibility

Once condition media and the potential range were optimized, four different carbon based SPEs (bare carbon, carbon nanotubes, carbon nanofibers, and graphene) were assessed. DPV measurements of a 10 mg L^−1^ PA, IB, and CF in acetate buffer at pH 5.5 were recorded. Table 1 summarizes the repeatability and reproducibility values calculated for PA, IB, and CF with each electrode. Repeatability was assessed using the same carbon based SPE unit for ten repetitive measurements yielding RSDs ranging from 2.2 to 11.0 % depending on both the considered analyte and the tested SPE. The reproducibility estimated from three different carbon based SPE units within a series of ten repetitive measurements produced RSDs ranging from 4.2 to 24.0% depending again on both the considered analyte and the tested SPE. In general terms, the repeatability and reproducibility values provided by SPGPHE are higher than those attained by the other carbon based SPEs, which could be attributed to the poor uniformity between the different SPGPHE units commercially obtained. Even so, all the obtained values are comparable to those described for the in-situ antimony film screen-printed electrode coated on different carbon substrates (bare carbon, graphene, carbon nanotubes and carbon nanofibers) [28] for Cd(II) and Pb(II) determination by differential pulse anodic stripping voltammetry (DPASV), or even better to those provided by SPCNFE for the determination of polyphenols by high performance liquid chromatography coupled to electrochemical detection [30].

On the other hand, all tested carbon based SPE could be used for a large set of measurements (more than 20) without loss of sensitivity enabling the voltammetric determination of PA, IB, and CF using the same SPE unit.

### 3.3. Sensitivity, Linearity, Limit of Detection (LOD), and Limit of Quantification (LOQ)

**Figure 1:**
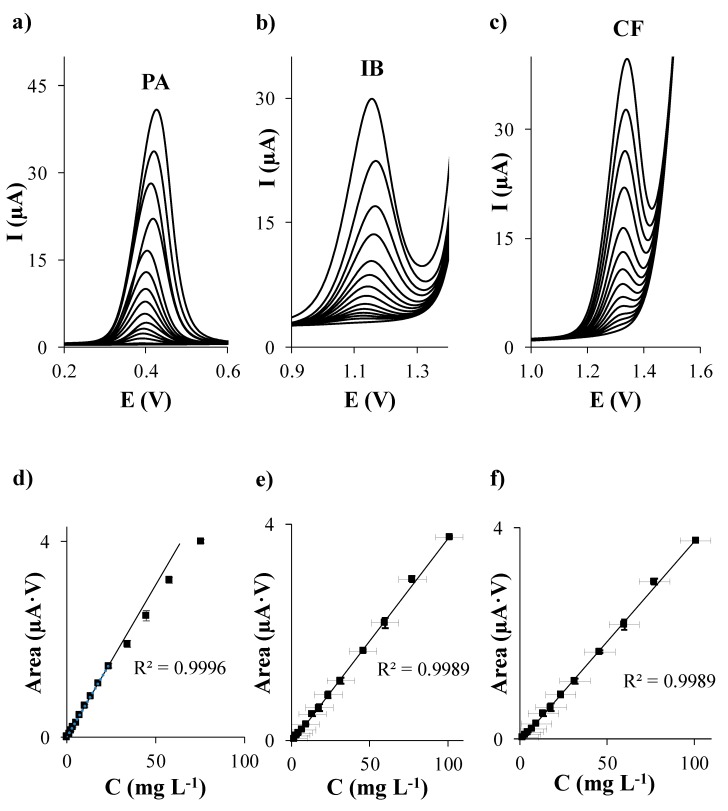
Separate differential pulse (DP) voltammograms of paracetamol (**a**), ibuprofen (**b**), and caffeine (**c**); and their respective calibration plots (**d**), (**e**), and (**f**) in 0.1 mol L^−1^ acetate buffer (pH 5.5) using a carbon screen-printed electrode (SPCE).

**Figure 2:**
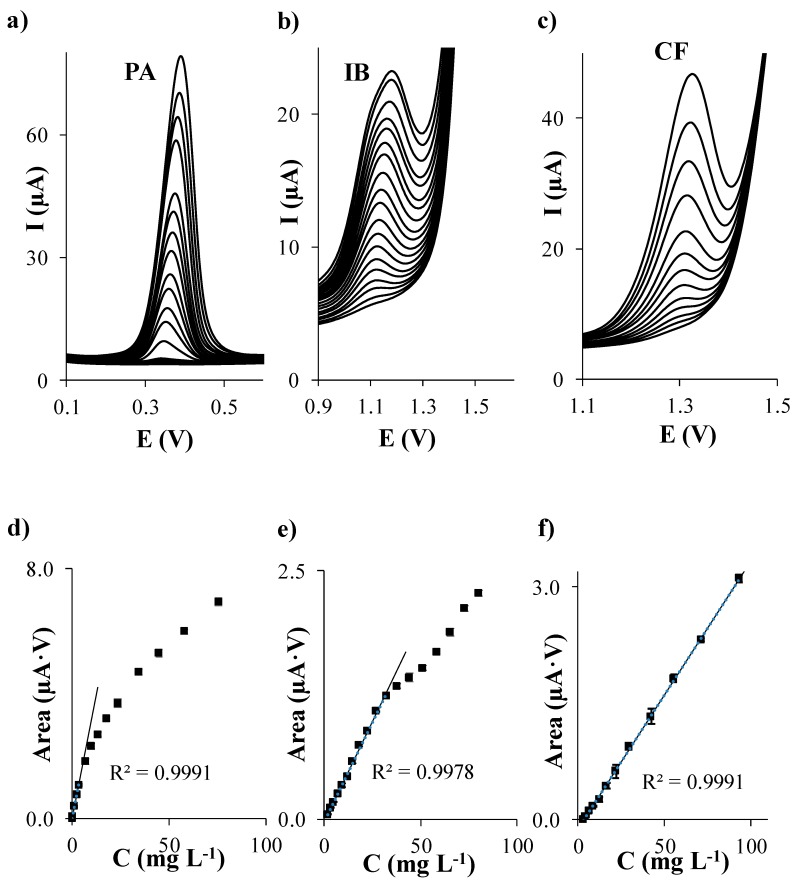
Separate differential pulse (DP) voltammograms of paracetamol (**a**), ibuprofen (**b**), and caffeine (**c**); and their respective calibration plots (**d**), (**e**) and (**f**) in 0.1 mol L^−1^ acetate buffer (pH 5.5) using a carbon nanotubes modified screen-printed electrode (SPCNTE).

**Figure 3:**
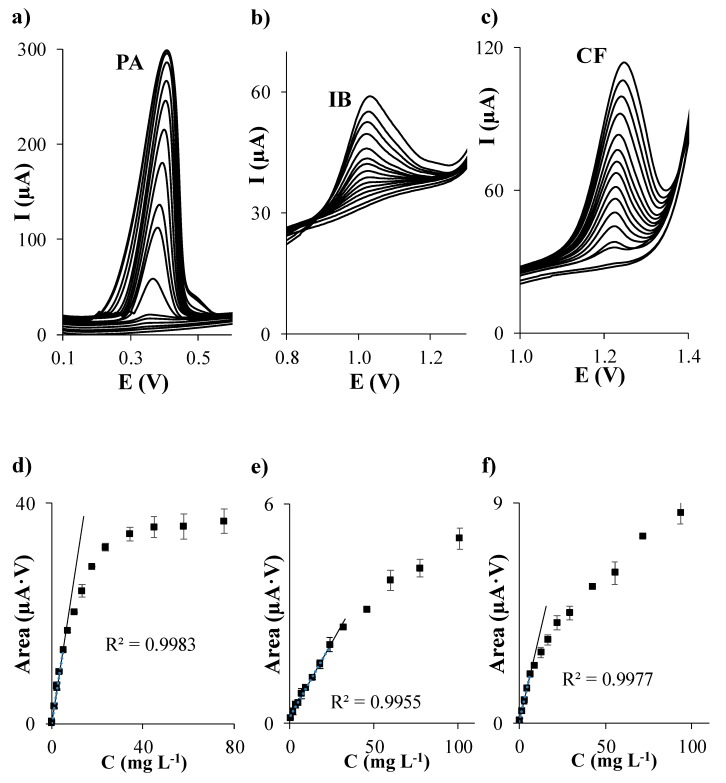
Separate differential pulse (DP) voltammograms of paracetamol (**a**), ibuprofen (**b**), and caffeine (**c**); and their respective calibration plots (**d**), (**e**) and (**f**) in 0.1 mol L^−1^ acetate buffer (pH 5.5) using a carbon nanofibers modified screen-printed electrode (SPCNFE).

**Figure 4:**
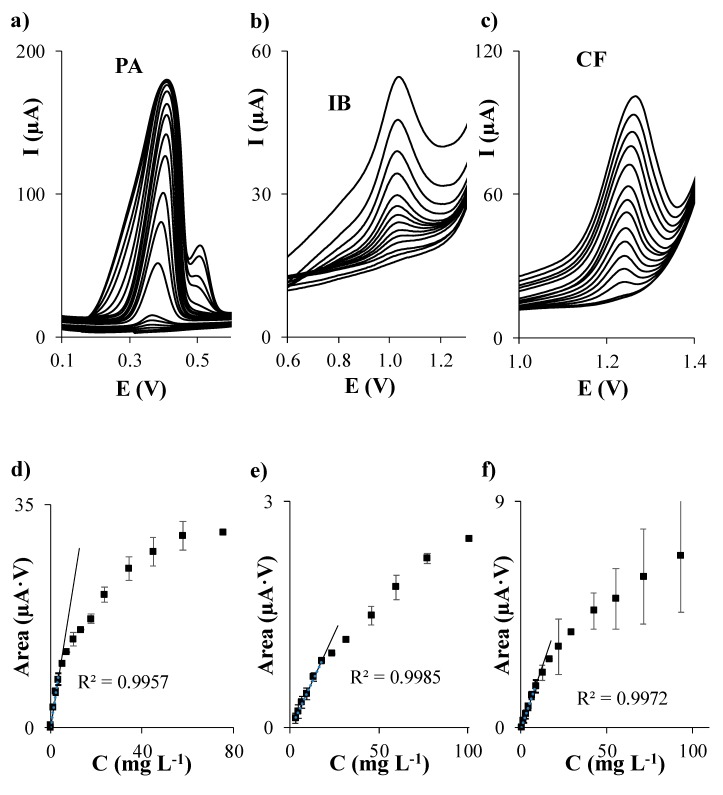
Separate differential pulse (DP) voltammograms of paracetamol (**a**), ibuprofen (**b**), and caffeine (**c**); and their respective calibration plots (**d**), (**e**), and (**f**) in 0.1 mol L^−1^ acetate buffer (pH 5.5) using a graphene screen-printed electrode (SPGPHE).

First of all, individual calibration of PA, IB, and CF in acetate buffer at pH 5.5 was performed on each considered carbon based SPE. The evolution of DPV signals of each analyte when the concentration of PA, IB, and CF individually increases is shown in Figure 1a–c, Figure 2a–c, Figure 3a–c, and Figure 4a–c using SPCE, SPCNTE, SPCNFE, and SPGPHE, respectively. In all cases, well-shaped peaks without indication of signal splitting were detected over the studied concentration range. Sensitivities, correlation coefficients, LODs, and linear ranges for PA, IB, and CF using SPCE, SPCNTE, SPCNFE, and SPGPHE are summarized in Table 2. Linear calibration plots were attained at the above established conditions by measuring fourteen increasing concentration of PA, IB, and CF ranging from 2 µg L^−1^ to 100 mg L^−1^ using SPCE (Figure 1d–f), SPCNTE (Figure 2d–f), SPCNFE (Figure 3d–f), and SPGPHE (Figure 4d–f). Sensitivities stated from the slopes of calibration plots ranged from 0.056 to 2.2 μA V mg^−1^ L for PA, from 0.0380 to 0.082 μA V mg^−1^ L for IB and from 0.0345 to 0.31 μA V mg^−1^ L for CF depending on the considered SPE. Overall, the highest sensitivities were provided by SPCNFE and SPGPHE.

The LOD and LOQ were assessed as 3 and 10 times, respectively, the standard deviation of the intercept over the slope of the calibration curve of the target analytes. The lowest value of the linear range was considered from the LOQ. The LOD of the analysis for the considered analytes in the four carbon based SPE varied from 0.1 to 0.6 mg L^−1^ for PA, from 0.6 to 1.9 mg L^−1^ for IB and from 0.4 to 1.4 mg L^−1^ for CF depending on the used SPE (Table 2) and the LOQ ranged from 0.3 to 2.0 mg L^−1^ for PA, from 1.9 to 6.3 mg L^−1^ for IB and from 1.2 to 4.8 mg L^−1^ for CF depending on the used SPE (Table 2).

Very good linear responses of the peak area versus concentration were achieved for all considered analytes up to a concentration level ranging from 3.5 to 57.8 mg L^−1^ for PA, from 17.8 to 100.9 mg L^−1^ for IB and from 6.4 to 93.3 mg L^−1^ for CF, depending, as stated before, on the considered SPE. Overall, the lowest LODs for the individual determination of PA, IB, and CF were achieved using SPCNTE and SPCNFE, which could be linked with the much larger active surface area that offers these modified carbon-based SPEs compared to the bare SPCE. However, the bare SPCE was the SPE that provided the wider linear range. Therefore, in view of the results obtained, it can be concluded that the optimal sensor for the determination of PA, IB, and/or CF at low concentrations, such as these detected in waters, would be the SPCNFE due to their high sensitivities and low LOQ values, and all further experiments were carried out using SPCNFE. Nevertheless, it should be highlighted that for the determination of high concentrations of PA, IB, or CF, such as these reported in pharmaceutical samples, the bare SPCE would be the best choice considering the lowest cost of the device and the wide linear range presented.

**Figure 5:**
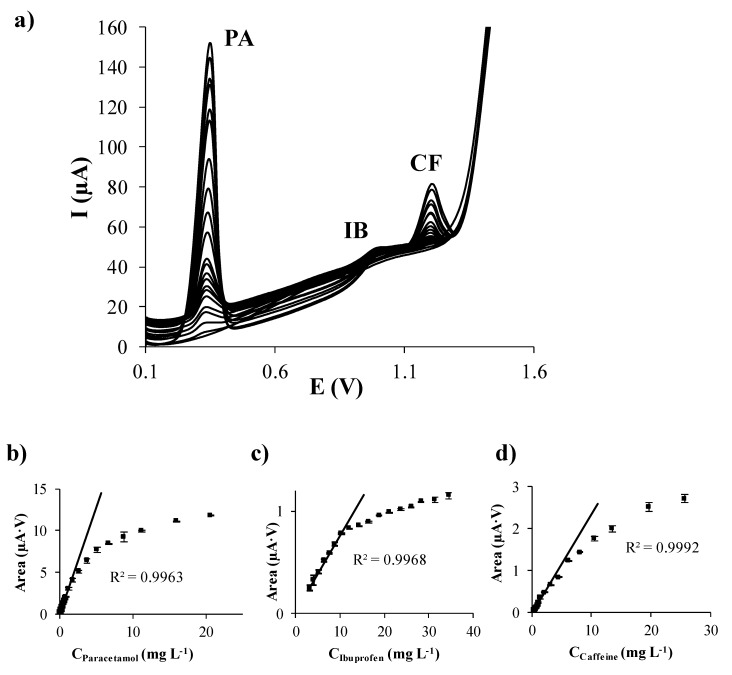
(**a**) differential pulse (DP) voltammograms of a mixture of paracetamol, ibuprofen and caffeine; and their respective calibration plots (**b**), (**c**) and (**d**) in 0.1 mol L^−1^ acetate buffer (pH 5.5) using a carbon nanofibers modified screen-printed electrode (SPCNFE).

In order to study if the coexistence of all the considered analytes influences the individual analytical performance of PA, IB, or CF, simultaneous calibration of PA, IB, and CF by DPV using SPCNFE was performed (Figure 5). Well-defined peaks without signal splitting were obtained for PA, IB, and CF on SPCNFE in the considered concentration range. Table 3 summarizes the calibration data for this simultaneous determination. Sensitivities obtained as the slope value of the simultaneous calibration plots of PA, IB, and CF were essentially the same as those provided in separate calibration, concluding that the coexistence of PA, IB, and CF does not affect their determination. In all cases, slightly lower LODs (0.03, 0.6 and 0.05 mg L^−1^ for PA, IB, and CF, respectively) and LOQs (0.09, 2.2 and 0.2 mg L^−1^ for PA, IB, and CF, respectively) than those achieved in separate calibration, were computed from these simultaneous calibration plots. Nevertheless, for all the considered analytes, narrower linear ranges were achieved, which is not a real problem when, as in this case, the analysis focuses on the determination of low concentrations of PA, IB, and CF. It should be mentioned that, to the best of our knowledge, only one preliminary study reported the individual determination of IB using a SPCNFE [20] with similar results to those obtained in this paper for this compound. However, it is worth noting that no previous data regarding the simultaneous voltammetric calibration of PA, IB, and CF neither using any SPEs nor with any other electrodes are accessible in the literature. Compared to previous reported results attained for the individual or the simultaneous determination of two of them using other electrodes (Table 4), e.g., multiwalled carbon nanotube modified basal plane pyrolytic graphite electrode by adsorptive stripping voltammetry [9], poly (4-vinylpyridine)/multiwalled carbon nanotubes modified glassy carbon electrode by DPV and CV [10], or carbon nanotube modified pyrolytic graphite electrode by CV or SWV [11] for the PA determination, the LODs obtained in this work are slightly higher. However, in comparison to other electrodes, such as carbon paste-multiwalled carbon nanotube composite electrode [17], poly(L-aspartic acid) modified glassy carbon electrode [18], or boron-doped diamond electrode [19] for IB determination by CV, DPV, SWV; gold nanoparticle-glassy carbon paste composite electrode [21], anthraquinone modified carbon paste electrode [22], or most of the electrodes considered in the review for CF determination by SWV, DPV, or stripping voltammetry [23]; nanogold modified indium tin oxide electrode [12], Nafion/TiO_2_–graphene modified glassy carbon electrode [13], C_60_-modified glassy carbon electrode [14], carbon ionic liquid electrode [15], or chitosan modified carbon paste electrode [16] for PA determination by CV, DPV, and SWV; HKUST-1 metal-organic framework-carbon nanofiber composite electrode for IB and diclofenac determination by CV [24]; boron-doped diamond electrode for the concurrent determination of PA and IB [25] or PA and CF [26] by DPV; and glassy carbon electrode for PA and phenobarbital determination by stripping voltammetry [27], the LODs achieved for the simultaneous determination of PA, IB, and CF by DPV using a SPCNFE are similar or even significantly better depending on the electrode considered. Moreover, it is worth noting that most of the sensors used for the determination of such analytes involved modifications of different complexity, whereas the proposed SPCNFE can be used, as it is commercially purchased without any previous modification or treatment, which greatly decreases the time dedicated to the electrode preparation, and with the additional advantages of screen-printed electrodes such as the low cost, disposable character, and the easy attachment to portable instrumentation facilitating the *in-situ* analysis.

### 3.4. Method Application

The applicability of the SPCNFE for the simultaneous determination of PA, IB, and CF by DPV was assessed by analyzing a tap water sample spiked with 1.63, 19.95, and 1.95 mg L^−1^ of PA, IB, and CF, respectively. The analytes were determined by means of the standard addition method. Then, DPV measurements following the above stated conditions were done including the additions of considered analytes. A complete replicate was measured using the same SPCNFE unit.

**Figure 6:**
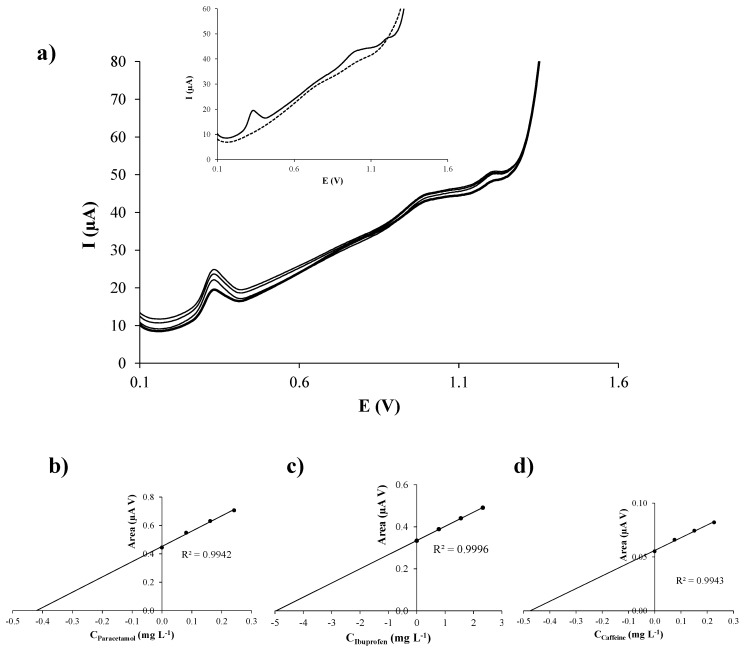
(**a**) differential pulse voltammetry (DPV) measurements in a tap water sample by using a carbon nanofibers modified screen-printed electrode (SPCNFE) in 0.1 mol L^−1^ acetate buffer (pH 5.5); and (**b**) paracetamol standard addition plot, (**c**) ibuprofen standard addition plot, and (**d**) caffeine standard addition plot.

Representative DP voltammograms obtained in the analysis of the spiked tap water sample using SPCNFE are presented in Figure 6a. Well−defined peaks for PA, IB, and CF were achieved. The calibration plots for PA, IB, and CF (Figure 6b–d respectively) illustrate the good correlations of the representative DPV measurement obtained on a SPCNFE. Table 5 shows the results of the analysis of three replicates of the tap water sample. As it can be seen, the concordance between PA, IB, and CF concentrations from the three replicates was quite good, particularly for IB. In all cases, the spiked and determined values of PA, IB, and CF concentrations were comparable and satisfactory recoveries of considered analytes in tap water (97.6 to 103.1%) were achieved (Table 5).

In order to test a real water sample and with potentially a high load of pharmaceutical residues, a hospital wastewater sample from Tunisia was carried out by both DPV using a SPCNFE and LC-MS/MS. A very good agreement between the different DPV replicates was achieved (RSD: 3.7%), as well as PA, could be detected. The concentration of PA determined by DPV (1.17 mg L^−1^) was very similar to that obtained by LC-MS/MS (1.27 mg L^−1^). It should be mentioned that IB and CF were not detected since the studied hospital wastewater sample did not contain IB and the concentration of CF was below the LOD obtained for the SPCNFE. However, the considered hospital wastewater sample contained other drugs such as sulfonamides, analgesics, anti-inflammatories, β-blockers, and anti-epileptics with a total concentration at levels of mg L^−1^ without signs of interference in the PA voltammetric signal. The good results obtained confirm the appropriateness of the proposed DPV method for the determination of such analytes in environmental samples even in the presence of other drugs. Moreover, it should be pointed out that this DPV method could also be extended to the analysis of environmental water samples with very low concentrations of the considered analytes (low μg L^−1^ or ng L^−1^) by prior preconcentration of analytes from large sample volume of water by solid phase extraction.

## 4. Conclusions

In this work, the analytical performance of four different screen-printed carbon-based electrodes (SPCE, SPCNTE, SPCNFE, and SPGPHE) was compared for the simultaneous determination of PA, IB, and CF by DPV. First of all, the potential range and condition media were optimized, achieving the best DPV response in 0.1 mol L^−1^ acetate buffer (pH 5.5) and scanning the potential from 0.1 to 1.75 V. At the abovementioned conditions, all the SPEs achieved LODs and LOQs at levels of mg L^−1^ for PA, IB, and CF. However, SPCNFE was selected as the optimal sensor for the determination of the considered analytes at low concentrations due to their highest sensitivity and LOQ values at very low mg L^−1^. Even if ultra-trace analysis (low μg L^−1^ or ng L^−1^) were required, the developed DPV method could be extended by previous preconcentration of analytes, for instance, by solid phase extraction. Moreover, it can be also concluded that the determination of each considered analyte is not affected by the presence of the other two compounds. Therefore, the good results obtained suggest that the DPV method using SPCNFE can be a suitable option to the more powerful yet expensive chromatographic-mass spectrometry methods for the determination of such analytes in environmental water samples. Furthermore, the SPCNFE can be used as commercially purchased without any previous modification, significantly decreasing the time devoted to the electrode preparation. Additional advantages of SPE include good reproducibility, low-cost, and the possibility to be connected to portable instrumentation.

Finally, the applicability of SPCNFE for the voltammetric analysis of real environmental samples was successfully demonstrated by the simultaneous determination of PA, IB, and CF in spiked tap water samples, in which good recoveries (103.1, 99.5, and 97.6% for PA, IB, and CF, respectively) and reproducibility (RSD of 8.93, 0.96, and 8.63% for PA, IB and CF, respectively) were achieved. In addition, the suitability of the DPV method using SPCNFE was also proved by the successful comparison of the results obtained from the determination of PA in a hospital wastewater sample achieved by LC-MS/MS.

## Figures and Tables

**Table 1:** Repeatability and reproducibility data for the determination of paracetamol, ibuprofen, and caffeine on bare carbon, carbon nanotubes, carbon nanofibers, and graphene (SPCE, SPCNTE, SPCNFE, and SPGPHE) at acetate buffer at pH 5.5.

Electrode	Paracetamol		Ibuprofen		Caffeine	
	Repeatability (RSD%)	Reproducibility (RSD%)	Repeatability (RSD%)	Reproducibility (RSD%)	Repeatability (RSD%)	Reproducibility (RSD%)
**SPCE**	2.2	4.4	5.2	5.5	4.2	6.5
**SPCNTE**	2.4	11.0	2.9	4.2	4.4	5.8
**SPCNFE**	3.2	5.1	4.1	4.6	4.7	10.2
**SPGPHE**	5.2	12.8	11.0	24.0	9.1	10.9

**Table 2:** Method performance for the individual determination of paracetamol, ibuprofen, and caffeine on bare carbon, carbon nanotubes, carbon nanofibers, and graphene (SPCE, SPCNTE, SPCNFE, and SPGPHE) at acetate buffer at pH 5.5. The standard deviations are denoted by parenthesis.

Electrode	Paracetamol				Ibuprofen				Caffeine			
	Sensitivity (μA V mg^−1^ L)	R^2^	Linear Range ^a^ (mg L^−1^)	LOD (mg L^−1^)	Sensitivity (μA V mg^−1^ L)	R^2^	Linear Range ^a^ (mg L^−1^)	LOD (mg L^−1^)	Sensitivity (μA V mg^−1^ L)	R^2^	Linear Range ^a^ (mg L^−1^)	LOD (mg L^−1^)
**SPCE**	0.056 (0.001)	0.999	2.0–57.8	0.6	0.0380 (0.0003)	0.999	3.8–100.9	1.1	0.0366 (0.0004)	0.997	4.8–93.3	1.4
**SPCNTE**	0.293 (0.005)	0.996	0.4–5.1	0.1	0.0404 (0.0005)	0.998	1.9–32.0	0.6	0.0345 (0.0003)	0.999	4.0–93.3	1.2
**SPCNFE**	2.66 (0.04)	0.998	0.3–5.1	0.1	0.082 (0.003)	0.996	4.0–23.6	1.2	0.31 (0.01)	0.998	1.2–6.4	0.4
**SPGPHE**	2.2 (0.1)	0.996	0.5–3.5	0.1	0.051 (0.003)	0.999	6.3–17.8	1.9	0.20 (0.01)	0.997	3.0–8.7	0.9

^a^ The lowest value of the linear range is the limit of quantification (LOQ).

**Table 3:** Method performance for the simultaneous determination of paracetamol, ibuprofen, and caffeine on carbon nanofibers modified screen-printed electrode (SPCNFE) at acetate buffer at pH 5.5. The standard deviations are denoted by parenthesis.

	Sensitivity (μA V mg^−1^ L)	R^2^	Linear Range ^a^ (mg L^−1^)	LOD (mg L^−1^)
**Paracetamol**	2.50 (0.05)	0.996	0.09–0.8	0.03
**Ibuprofen**	0.074 (0.002)	0.997	2.2–10.2	0.6
**Caffeine**	0.24 (0.01)	0.999	0.2–1.1	0.05

^a^ The lowest value of the linear range was the limit of quantification (LOQ).

**Table 4:** Summary of voltammetric methods published so far for the determination of paracetamol, ibuprofen, and caffeine.

Electrode	Technique	Analyte	LOD (mg L^−1^)	Application	Ref.
Multiwalled carbon nanotube modified basal plane pyrolytic graphite electrode	AdSV	PA	0.002	Drugs	[9]
Poly (4-vinylpyridine)/multiwalled carbon nanotubes modified glassy carbon electrode	DPV	PA	0.0003	Drugs, urine	[10]
Carbon nanotube modified pyrolytic graphite electrode	CV SWV	PA	0.0004	Drugs, urine	[11]
Nanogold modified indium tin oxide electrode	DPV	PA	0.03	Drugs	[12]
Nafion/TiO_2_–graphene modified glassy carbon electrode	CV DPV	PA	0.03	Drugs	[13]
C_60_-modified glassy carbon electrode	DPV	PA	7.6	Drugs, urine	[14]
Carbon ionic liquid electrode	CV DPV	PA	0.05	Drugs, urine	[15]
Chitosan modified carbon paste electrode	CV SWV	PA	0.08	Water samples, drugs, urine	[16]
Carbon paste-multiwalled carbon nanotube composite electrode	DPV	IB	0.6	Drugs	[17]
Poly(L-aspartic acid) modified glassy carbon electrode	CV SWV	IB	0.03	Drugs, urine	[18]
Boron-doped diamond electrode	CV DPV	IB	0.8	Drugs	[19]
Screen-printed carbon electrode modified with carbon nanofibers	CV DPV	IB	0.05	Drugs	[20]
Gold nanoparticle-glassy carbon paste composite electrode	DPV	CF	0.2	Beverages	[21]
Anthraquinone modified carbon paste electrode	CV SWV	CF	0.02	Drugs	[22]
All the electrodes cited therein	Various	CF	9 × 10^−5^–47.4	Drugs, urine, serum, beverages	[23]^(^*^)^
HKUST-1 metal-organic framework-carbon nanofiber composite electrode	CV	IB, diclofenac	0.02 (IB)	Water samples	[24]
Boron-doped diamond electrode	DPV	PA, IB	1.1 (PA) 0.8 (IB)	Drugs	[25]
Boron-doped diamond electrode	SWV DPV	PA, CF	0.07 (PA) 0.007 (CF)	Drugs	[26]
Glassy carbon electrode	DPSV	PA, phenobarbital	0.04 (PA)	Drugs	[27]
Screen-printed carbon electrode modified with carbon nanofibers.	DPV	PA, IB, CF	0.03 (PA) 0.6 (IB) 0.05 (CF)	Water samples	This work

^(^*^)^ Reference 23 corresponds to a review about determination of caffeine. AdSV: adsorptive stripping voltammetry, DPV: differential pulse voltammetry, SWV: square wave voltammetry, CV: cyclic voltammetry, DPSV: differential pulse stripping voltammetry; PA: paracetamol, IB: ibuprofen, CF: caffeine.

**Table 5:** Total concentrations of paracetamol, ibuprofen and caffeine determined in spiked tap water by differential pulse voltammetry (DPV) on carbon nanofibers modified screen-printed electrodes (SPCNFE) by standard addition quantification method.

	Paracetamol	Ibuprofen	Caffeine
**C_determined_ (mg L^−1^)**	1.7 (0.2)	19.8 (0.2)	1.9 (0.2)
**RSD (%)**	8.93	0.96	8.63
**Relative error (%)**	3.1	0.5	2.4
**Recovery (%)**	103.1	99.5	97.6

n = 3 for RSD (%).

## Supplementary Materials

The following are available online at https://www.mdpi.com/1424-8220/19/18/4039/s1, Figure S1: DPV measurements of 20 mg L−1 PA, IB and CF solutions performed in the presence of different buffers: (a) 0.05 mol L−1 sulphuric acid (pH 1); (b) 0.1 mol L−1 ammonia buffer (pH 8.6); (c) 0.1 mol L−1 maleate buffer (pH 6.8); (d) 0.1 mol L−1 phosphate buffer (pH 7.4); and (e) 0.1 mol L-1 acetate buffer (pH 4.5)., Figure S2: DPV measurements of 20 mg L^−1^ PA, IB and CF solutions performed in 0.1 mol L-1 acetate buffer at different pHs: (a) pH 4.0; (b) pH 4.5; (c) pH 5.0; (d) pH 5.5; and (e) pH 6.0.
