# Supplementary material for: Commercial Screen-Printed Electrodes Based on Carbon Nanomaterials for a Fast and Cost-Effective Voltammetric Determination of Paracetamol, Ibuprofen and Caffeine in Water Samples
Source: Sensors (Basel). 2019 Sep 19;19(18):4039. doi: 10.3390/s19184039 (PMC6767221; doi:10.3390/s19184039)
Supplement: Supplementary file 1 [file sensors-19-04039-s001.pdf]

# Commercial screen-printed electrodes based on carbon nanomaterials for a fast and cost-effective voltammetric determination of paracetamol, ibuprofen and caffeine in water samples

**Núria Serrano [1,*], Òscar Castilla [1], Cristina Ariño [1,2], Maria Silvia Díaz-Cruz [3] and José Manuel Díaz-Cruz [1,2]**

[1] Department of Chemical Engineering and Analytical Chemistry, University of Barcelona. Martí i Franquès 1-11, E08028 Barcelona, Spain.
[2] Institut de Recerca de l'Aigua (IdRA), University of Barcelona.
[3] Department of Environmental Chemistry, Institute of Environmental Assessment and Water Research (IDAEA), Spanish Council of Scientific Research (CSIC), Jordi Girona 18-26, 08034-Barcelona, Spain.
* Correspondence: nuria.serrano@ub.edu; Tel.: 34-93-402-1277

Received: date; Accepted: date; Published: date

## Supporting Information

This supplementary section provides further description of the optimization of condition media and the potential range related to the simultaneous determination of paracetamol (PA), ibuprofen (IB) and caffeine (CF) by DPV using the conventional SPCE as a carbon based SPE model.

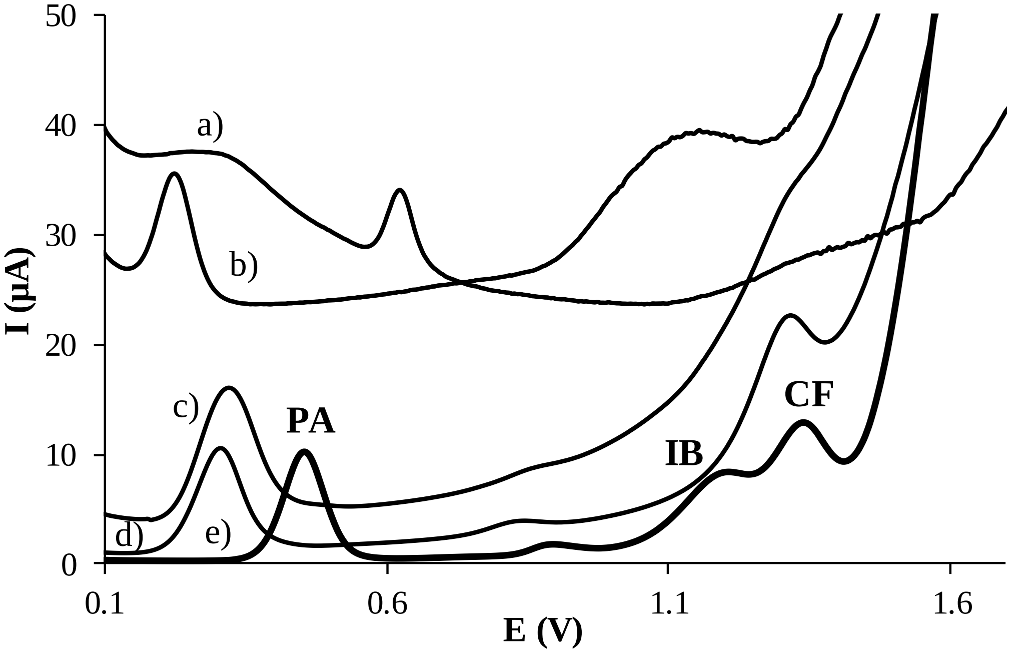


**Fig. S1.** DPV measurements of 20 mg $L^{-1}$ PA, IB and CF solutions performed in the presence of different buffers: a) 0.05 mol $L^{-1}$ sulphuric acid (pH 1); b) 0.1 mol $L^{-1}$ ammonia buffer (pH 8.6); c) 0.1 mol $L^{-1}$ maleate buffer (pH 6.8); d) 0.1 mol $L^{-1}$ phosphate buffer (pH 7.4); and e) 0.1 mol $L^{-1}$ acetate buffer (pH 4.5).

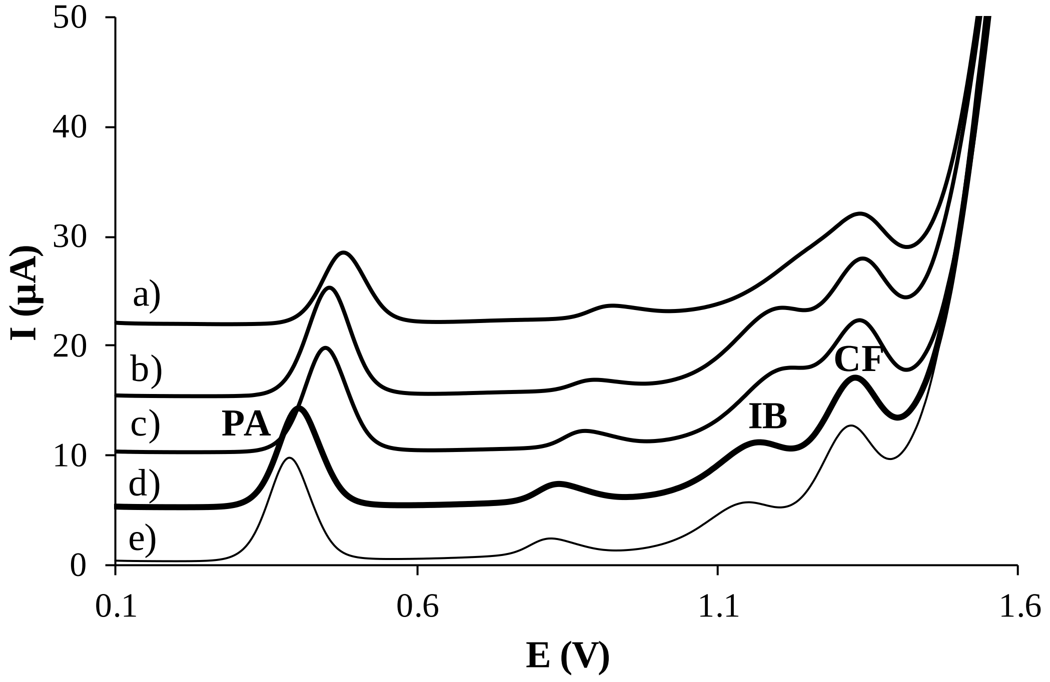


**Fig. S2.** DPV measurements of 20 mg $L^{-1}$ PA, IB and CF solutions performed in 0.1 mol $L^{-1}$ acetate buffer at different pHs: a) pH 4.0; b) pH 4.5; c) pH 5.0; d) pH 5.5; and e) pH 6.0.
